# Supplementary figures and images for: An Ultrasensitive Genetically Encoded Voltage Indicator Uncovers the Electrical Activity of Non‐Excitable Cells
Source: Adv Sci (Weinh). 2024 Mar 25;11(20):2307938. doi: 10.1002/advs.202307938 (PMC11132041; doi:10.1002/advs.202307938)

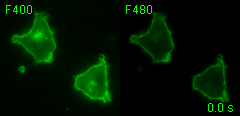

Supplement: Supplementary file 2 — Supplemental Video 1 [file ADVS-11-2307938-s003.gif]

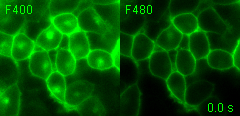

Supplement: Supplementary file 3 — Supplemental Video 2 [file ADVS-11-2307938-s002.gif]
